# The network structure of ego depletion in Chinese male young adults

**DOI:** 10.3389/fpsyg.2023.1102624

**Published:** 2023-05-17

**Authors:** Junji Ying, Lei Ren, Jiaxi Zhang, Yue Zhou, Xiaofang Zhang, Wei Xiao, Xufeng Liu

**Affiliations:** ^1^Department of Military Medical Psychology, Air Force Medical University, Xian, China; ^2^Xi'an Research Institute of High Technology, Xi’an, China; ^3^Institute of Social Technology, Suranaree University of Technology, Nakhon Ratchasima, Thailand

**Keywords:** ego depletion, self-control, network analysis, node centrality, predictability

## Abstract

Ego depletion refers to the state of low self-control ability as defined by the limited resource model of self-control. The ego depletion aftereffects scale (EDA-S) is a relatively mature tool for evaluating ego depletion. However, the internal structure of EDA-S is not clear. A deeper understanding of its internal structure, especially the core variables, is required to design better interventions to improve people’s ego depletion outcomes and self-control. In the present study, we estimated an unregularized partial correlation network of ego depletion in a sample of 499 male young adults in China, who participated in the EDA-S test, and calculated the centrality index. The results showed that all nodes in the ego depletion network were positively correlated. The five strongest edges were between somatic distress and fatigue, emotional regulation disorder and social withdrawal, work burnout and low self-efficacy, low adherence and low self-efficacy, and fatigue and low processing fluency. Fatigue, low self-efficacy, and emotional regulation disorder had the highest strength centrality, indicating that these three variables may play an important role in the network of ego depletion. This study conceptualizes ego depletion from the perspective of networks in order to provide potential targets for related interventions and insights for future studies.

## Introduction

People have to follow several rules in their everyday life and are required to exercise tremendous self-control. However, too much self-control leads to the rapid consumption of self-control resources, resulting in poor performance in making prudent decisions, overcoming difficulties, and resisting temptations. A state of ego depletion resulting from the failure to exert self-control is observed in emotional difficulties, addiction, crime, violence, underachievement, money problems, unwanted pregnancy, sexually transmitted diseases, obesity, eating disorders, substance abuse, prejudice, relationship problems, and more (Baumeister et al., 2018, 2019). (Baumeister et al. 2018, 2019) believe that low self-control is a genuine causal factor in many of these problems. The nature of ego depletion is a low self-control state. Recently, some studies have focused on differences in ego depletion among individuals based on their self-control ability. For example, why are some people more likely to give up difficult, unsolvable tasks or painful ice bucket tasks? Why are some people more likely to overeat or be more aggressive in life, even though they know it is wrong? Tangney et al. (2004) developed the theory of trait self-control and state ego depletion to describe such individual differences.

### Ego depletion and its assessment

The ego depletion model defines ego depletion as a series of behaviors that result in poor self-control at the physiological, cognitive, emotional, and behavioral levels. Researchers have attempted to measure the degree of ego depletion in individuals in various ways. In several studies, a number of tasks were employed to assess the extent of ego depletion, such as the Stroop task, Go/Nogo task, Ice Bucket task, and No-solution puzzle (Baumeister et al., 1998). Real-life behaviors, such as how much you smoke or drink, how many snacks you eat, and how many times you stop reading or exercising, also indicate the degree of ego depletion (Baumeister et al., 1998). However, the extent to which these indicators reflect ego depletion is unclear; for example, the performance in the Stroop task may not only indicates ego depletion but also learning ability.

In an attempt to measure the degree of ego depletion, various states of ego depletion have been studied, including emotional regulation disorders, social withdrawal, low adherence, and low self-efficacy. Muraven et al. (1998) used thought suppression tasks to deplete participants’ self-control resources. They found that participants whose self-control resources were depleted had difficulty keeping themselves from laughing during the subsequent observation of humorous movies. This suggests that ego depletion makes the suppression of emotional expressions particularly difficult. Studies have shown that individuals in a state of ego depletion are less able to disguise their negative traits (Bertrams et al., 2010), are more likely to engage in socially disruptive behaviors such as aggression (Barlett et al., 2016) and cheating (Mead et al., 2009), and are less likely to practice prosocial behavior (DeBono et al., 2011). Furthermore, individuals with ego depletion are less inclined to adhere to a consistent physical exercise routine, struggle with resisting the temptation to overeat (Inzlicht and Kang, 2010), tend to abandon their wellness plans prematurely due to the fear of failure (Martin and Bray, 2010), and are less likely to sustain a healthy lifestyle. In addition, individuals who experience ego depletion exhibit cognitive biases, tend to underestimate their abilities, have more pessimistic expectations about the future (Fischer et al., 2007), and feel less in control of the future.

The difference in ego depletion can reflect the individual difference in self-control ability to some extent. However, the exact relationship between ego depletion and self-control is not clear. There is a causal relationship, hierarchy, and centrality among the different states of ego depletion, which needs to be sorted out and integrated using new methods. Moreover, if ego depletion has a stable central dimension, it may be the key to linking other dimensions and may provide a new target for future interventions and ideas for training.

### The state self-control scale

Ego depletion is a phenomenon that the ability of control decreases due to the consumption of control resources while performing tasks that require self-control. The level of self-control is an important index to measure the degree of ego depletion. The evaluation of state self-control can directly reflect the level of individual ego depletion. The existing measures for evaluating state self-control include the following: 1. Self-regulation Questionnaire, compiled by Brown et al. (1999), can investigate individuals’ self-regulatory strengths and challenges can help identify their abilities and strategies for coping with specific problems in different situations; 2. State Self-control Capacity Scale, compiled by Twenge et al. (2004), includes 25 items to investigate the state of self-control that focused on ego depletion. Based on this scale, (Baumeister et al. 2018, 2019) also developed a brief scale for convenient use; 3. Self-regulatory Fatigue Scale, compiled by Nes et al. (2013), can assess the fatigue state of individuals due to adjusting resource consumption while performing tasks requiring self-control. The scale consists of 18 questions, covering three aspects of cognition, emotion and behavior, which can assess the degree of self-regulation fatigue.

The above scales have some limitations, which make them not suitable for the network analysis of ego depletion aftereffects in this study. The Self-regulation Questionnaire is not based on the ego depletion model. The State Self-control Capacity Scale is not publicly published. Baumeister et al.’s brief scale included three items, and some additional important dimensions were not included. The Self-regulatory Fatigue Scale is designed specifically for groups with chronic disease. Tang et al. (2016) developed the “ego depletion aftereffects scale (EDA-S),” which consists of 38 items, including nine factors, such as difficulty in emotion regulation, social withdrawal, and low self-efficacy. EDA-S is designed strictly on the basis of ego depletion model, and is dimensionally comprehensive, so it is suitable for network analysis of ego depletion for young adults.

### Network analysis

The basic assumption of the mainstream latent variable model is that there is a potential variable represented by its index, such as ego depletion, and the others are observational variables, such as fatigue and low self-efficacy. It follows that the observed variables are not directly related to each other, and their covariances are all due to the common influence of the latent variable. This phenomenon is fully explained by the shared potential variables, indicating that the observed variables have local independence, and the relationship among these observed variables is not the focus of the model.

Network analysis is an innovative statistical analysis method, which is driven by data and does not depend on the prior hypothesis involving variables to carry out a mathematical analysis and visual representation of the relationship among variables (Galderisi et al., 2018). Network analysis provides a new way to conceptualize psychological constructs, assuming that psychological constructs are a complex system phenomenon caused by the interaction of their components (Borsboom, 2017). In this case, the components are not passive indicators reflecting the concept itself, but rather are indicators playing an active role in the process of the emergence of the concept. Therefore, considering the complexity of ego depletion, it is reasonable and feasible to regard it as a complex system phenomenon caused by the interaction of different dimensions. Furthermore, network analysis can provide centrality and predictability for each node in the network to evaluate the degree of importance and controllability of each node. When activated, a node with high centrality is likely to propagate the activation to the whole network by connecting the edges of other nodes (Hofmann et al., 2016). This provides an important potential target for related interventions (Fried et al., 2016). Recently, a growing number of studies have used network analysis to explore psychological constructs such as empathy (Briganti et al., 2018), stigma (Wei et al., 2020), decision-making (Peng et al., 2020), burnout (Wu et al., 2021), personality (Costantini et al., 2015), anxiety (Beard et al., 2016), depression (Fried et al., 2016), and post-traumatic stress disorder (Mcnally et al., 2015).

### Aim of the study

Although EDA-S is a relatively mature tool for assessing ego depletion, a deeper exploration of its internal structure and core dimensions is useful and necessary for future studies and related interventions. In the present study, we use network analysis to investigate how the different dimensions of ego depletion relate to each other in Chinese male young adults. We are particularly interested in the strength and predictability of each variable. Although previous studies have attempted to investigate the factor structure of ego depletion, they have rarely expected or suggested the existence of central factors. If the central factors can be extracted, the concept of ego depletion can be better understood, and it is expected to provide new and more efficient methods for future training interventions.

## Materials and methods

### Participants

Using the convenient sampling method, during the final exam month, 523 participants were recruited from six majors of three grades in a public university in Xi’an. The selection of disciplines for different grades and majors avoids the homogeneity of the participants. The pressure students face on the final exam also helps us to collect samples of different levels of ego depletion. Of the 523 participants, 16 dropped out of the EDA-S test and did not complete all of the items, and 8 answered the items according to explicit rules (for example, select one answer for all items). The remaining 499 participants were all men aged 19 to 25, with an average age of 21.01 years (SD = 1.587). The tests were administered by a psychology teacher and six graduate students who had received training in psychology. They informed the participants of the study’s objectives and implications and shared that the answers were not marked as true or false. The data were collected in a college classroom on the spot after all participants signed informed consent forms. The students were informed that they would receive research credits in exchange for their participation, which also guaranteed that they would answer the questions truthfully.

### Ego depletion aftereffects scale

The EDA-S was compiled by Yicheng Tang and colleagues in 2016. The scale consists of 38 items divided into nine factors: emotion regulation disorder, social withdrawal, low self-efficacy, working memory loss, low processing fluency, work burnout, fatigue, somatic distress, and low adherence. The participants chose a realistic description based on their actual experience in the last 2 weeks. Choosing between 1 (very inconsistent) and 5 (very consistent), the higher the score, the greater the degree of ego depletion. Somatic distress (SD) included eight items, α = 0.887, e.g., I feel chills or fever; Fatigue (FA) included six items, α = 0.886, e.g., I feel more tired than before; Low processing fluency (LPF) included five items, α = 0.869, e.g., My reading speed is not as fast as it used to be; Work burnout (WB) included three items, α = 0.889, e.g., I feel bored with study or work; Working memory loss (WML) included four items, α = 0.841, e.g., I often forget what to do in the middle of things now; Emotional regulation disorder (ERD) included three items, α = 0.851, e.g., I feel more difficult to control my temper than before; Social withdrawal (SW) included three items, α = 0.764, e.g., I am less likely to interact with people than before; Low adherence (LA) included three items, α = 0.727, e.g., I am more reluctant to take part in physical exercise than before; Low self-efficacy (LSE) included three items, α = 0.86, e.g., I have no confidence in my ability to work or study.

### Network analysis

The networks were estimated *via* Gaussian graphical models (GGMs; Epskamp et al., 2018a). GGMs are undirected networks in which the edges represent partial correlations between two nodes, after conditioning on all other nodes in the network. The nonparametric Spearman rho correlations were used when estimating the network structure, as recommended by Epskamp and Fried (2018). Due to the small number of variables (nine variables) but more samples (499 individuals), we adopted unregularized model selection rather than regularization techniques commonly used in estimating GGMs (Williams and Rast, 2020). The visualization of networks was derived from the Fruchterman–Reingold algorithm, which locates nodes with stronger and more numerous connections near the center of the network and weakly associated nodes on the periphery (Fruchterman and Reingold, 1991). In the visualized networks, blue edges represent positive correlations, and red edges represent negative correlations. Thicker edges mean stronger correlations between the nodes. The networks were constructed and visualized using the R-package *qgraph* (Epskamp et al., 2012).

Recent studies have shown that strength is the most reliable centrality index, and the centrality indices of betweenness and closeness are unsuitable for assessing the importance of nodes in psychological networks (Bringmann et al., 2019). Therefore, we calculated strength centrality for each node to evaluate and quantify their relative importance in the entire network using the R-package *qgraph* (Epskamp et al., 2012). Node strength is defined as the sum of the absolute value of the edge weights attached to a given node. A higher value of strength indicates greater importance in the network. In addition, we computed the predictability of each node by using the R-package *mgm* (Haslbeck and Fried, 2017). Predictability is defined as the variance of a node which is explained by all its neighboring nodes and can characterize the controllability of the node (Ren et al., 2021).

We examined the robustness of network by using the R-package *bootnet* (Epskamp et al., 2018b). First, the accuracy of edge weight was evaluated by calculating 95% confidence intervals (CI) using a non-parametric bootstrap approach (2000 bootstrap samples). Second, the stability of node strength was evaluated by computing correlation stability (CS) coefficient, using a case-dropping bootstrap approach. The value of CS-coefficient should not be below 0.25 and preferably should be above 0.5. Third, bootstrapped difference test (2000 bootstrap samples and α = 0.05) for edge weight and node strength were performed to evaluate whether two edge weights or two node strengths differ significantly from one another.

## Results

Descriptive statistics: The mean age of the 499 students was 21.01 ± 1.587 years (mean ± SD; range: 19 to 25). All the participants were male, including sole offspring, non-sole offspring, urban residents, and rural residents. Table 1 shows the abbreviation, mean scores, standard deviations, node strength (*Z*-scored), and predictability for each dimension of the EDA-S. Supplementary Table S1 shows the nonparametric Spearman rho correlation matrix of these dimensions (see Supplementary material).

**Table 1:** Abbreviation, mean scores, standard deviations, node strength (Z-scored), and predictability for each dimension of the EDA-S.

Dimensions (Abbreviation)	*M*	*SD*	Str	Pre
Somatic distress (SD)	15.31	7.08	−1.42	0.53
Fatigue (FA)	16.43	6.51	1.08	0.59
Low processing fluency (LPF)	11.70	4.99	0.23	0.56
Work burnout (WB)	6.49	3.24	0.48	0.56
Working memory loss (WML)	8.15	3.73	−1.82	0.39
Emotion regulation disorder (ERD)	5.59	2.90	0.65	0.60
Social withdrawal (SW)	5.57	2.72	0.45	0.60
Low adherence (LA)	6.22	2.77	−0.35	0.54
Low self-efficacy (LSE)	6.41	3.04	0.70	0.55

EDA-S, Ego depletion aftereffects scale; M, Mean; SD, Standard deviation; St, Node strength; Pre, Predictability.

**Figure 1:**
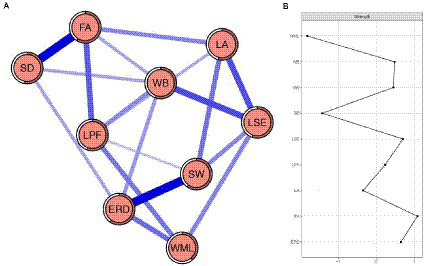
**(A)** EDS-A network. Somatic distress (SD), fatigue (FA), low processing fluency (LPF), work burnout (WB), working memory loss (WML), emotional regulation disorder (ERD), social withdrawal (SW), low adherence (LA), and low self-efficacy (LSE). The thickness of the edges represents the degree of correlation. The blue edge represents a positive correlation, and the red edge represents a negative correlation. The rings around the nodes describe its predictability. **(B)** Strength of *Z*-scores of each node in EDS-A network. Somatic distress (SD), fatigue (FA), low processing fluency (LPF), work burnout (WB), working memory loss (WML), emotional regulation disorder (ERD), social withdrawal (SW), low adherence (LA), and low self-efficacy (LSE).

Network structure: The network of EDA-S is shown in Figure 1A. The following network characteristics are observed. First, 18 of the 36 possible edges (50%) are not zero, and all the edges are positive. Second, in the final network, the five strongest edges are between “Somatic distress” and “Fatigue,” “Emotion regulation disorder” and “Social withdrawal,” “Work burnout” and “Low self-efficacy,” “Low adherence” and “Low self-efficacy,” and “Fatigue” and “Low processing fluency.” Bootstrapped 95% confidence interval indicating the accuracy of edge weight was relatively reliable and accurate (see Supplementary Figure S1 in the Supplementary material). Moreover, in the present network, bootstrapped difference test for edge weight indicates that the two strongest edge weights are significantly different from about 94% (16/17) of the other edge weights (see Supplementary Figure S2 in the Supplementary material). Third, node predictability is visualized as a circle around the node in Figure 1A. The value of node predictability ranges from 39 to 60%, with an average of 55%. This indicates that, on average, 55% of the variance of nodes in the current network can be explained by their neighboring nodes (see Table 1).

The strength centrality of the nine dimensions is shown in Figure 1B. Dimensions “Fatigue,” “Low self-efficacy” and “Emotion regulation disorder” have the highest strength centrality, indicating that these three dimensions have the most associations in the present network. Dimensions “Working memory loss” and “Somatic distress” have the lowest strength centrality, indicating that these two dimensions are the least associated in the current network. The correlation stability coefficient of node strength centrality is 0.44, indicating that the estimations of node strength centrality meet the requirements (see Supplementary Figure S3 in the Supplementary material). The results of bootstrapped difference test for node strength centrality are shown in Supplementary Figure S4 (see Supplementary material).

## Discussion

This study applies network analysis to EDA-S. This is the first time that network analysis has been applied to evaluate ego depletion. Network theory provides an alternative to conceptualizing the psychological structure as an interacting system involving various components, which actively participates in the emergence of the concept, rather than being a passive indicator of the network structure (Fried et al., 2016). Therefore, it is reasonable to regard ego depletion as an interactive system, consisting of different representations of ego depletion and providing a new perspective for describing and understanding ego depletion. Network analysis is a relevant tool that can reveal the psychological structure of ego depletion. It can mathematically analyze and visually show the relationship between complex variables, independent of the prior hypothesis of causality between variables (Dalege et al., 2017). In particular, network analysis can be used to examine the strength of the core variables in the network, compared to mere correlational approaches.

The primary goal of this study is to explore the relationship between the components of ego depletion. Second, the centrality index is used to quantify the importance of each component in the current network, providing insights for future research and potential targets for related interventions. Several experiments have shown that self-control can be improved through teaching and training. A comprehensive investigation into the internal structure and core dimensions of ego depletion can help design interventions to improve people’s ego depletion outcomes and self-control.

The results of the present study show that all nodes of the ego depletion network are positively correlated. The edge results show that “Emotion regulation disorder” and “Social withdrawal” are the most closely related. Close associations are also found between “Somatic distress” and “Fatigue,” “Work burnout” and “Low self-efficacy,” “Low adherence” and “Low self-efficacy” and “Fatigue” and “Low processing fluency,” which are basically consistent with those of previous simple correlation research methods (Tang et al., 2016b). Strong partial correlation suggests that the two connected nodes may co-occur highly. For the relationship between “Emotion regulation disorder” and “Social withdrawal,” emotional regulation disorder can lead to individual emotional fluctuations, psychological instability, thus increasing the possibility of social withdrawal. Emotion regulation disorder may affect the individual’s psychological state and emotional stability, making it difficult for individuals to cope with various pressures in the environment, and thus withdraw from social activities halfway. Social withdrawal may also exacerbate an individual’s dysregulation of emotions. Social activity is an important way for individuals to gain social support and satisfaction. Social withdrawal can lead individuals to withdraw from social networks and reduce the sources of social support, lead to excessive emotional tension, anxiety and fatigue and other negative emotions, which aggravate the emotional regulation barriers. The two-way relationship between the two is also supported by previous studies (Desjardins et al., 2019). They may activate and reinforce each other, thus making the vicious circle stable and difficult to break. Based on the above analysis, in order to effectively reduce ego depletion, it is suggested to strengthen the intervention of individual emotion regulation, increase emotional stability, simultaneously encourage them to participate in social activities, expand social networks, and increase the sources of social support, moderates the interaction between emotion regulation disorder and social withdrawal. The strong partial correlation between “Somatic distress” and “Fatigue,” means that fatigue may be an important factor leading to somatic distress. First of all, fatigue may lead to internal dysfunction of the body, which can lead to somatic distress. Second, when people are tired, they may choose unhealthy ego depletion behaviors, such as sitting for long periods of time playing video games or using smartphones, which can lead to somatic distress. Finally, long-term fatigue may cause a series of physical and psychological problems, such as anxiety, depression and so on, thus affecting physical health. Many studies have found that there is a significant positive correlation between the degree of fatigue and somatic distress symptoms, especially in chronic diseases (Valentine et al., 2022). For the relationship between “Low adherence” and “Low self-efficacy,"research found that low self-efficacy may affect a person’s requirements for persistence (Yeager et al., 2014). If a person is confident in his or her abilities, he or she will be more likely to perform tasks that are consistent. On the contrary, if a person thinks he cannot do something well, then he will be more likely to give up the task. Therefore, self-efficacy may be an important factor affecting persistence. On the other hand, low adherence may also affect self-efficacy, making people doubt and distrust their abilities. When a person in the face of difficulties, if he does not have enough patience and perseverance, then he is difficult to achieve success in action, then his sense of self-efficacy is difficult to improve or confirm. Low adherence may lead to self-doubt, which can reduce self-efficacy. For the relationship between “Work burnout” and “Low self-efficacy,” low self-efficacy will lead to work burnout, which is a kind of psychological and behavioral state, it will reduce the motivation and ability to perform tasks, conditions that force people to become tired, bored, give up easily, and lack energy. When individuals have a low sense of self-efficacy, they will feel that their ability is very low, it is difficult to complete the task. Therefore, they feel that their work and study efforts are useless, it is easy to appear the phenomenon of burnout. In addition, the negative factors of self-perception may lead to the lack of self-adjustment strategies, lack of self-awareness and work-study methods, which will further reduce the work and study performance. Several studies have shown that low self-efficacy is significantly associated with work burnout, and one study of firefighters found that self-efficacy moderates the relationship between stress and work burnout, suggesting that people should pay particular attention to developing self-efficacy, make it an important part of your work burnout prevention program. For the relationship between “Fatigue” and “Low processing fluency,” the relationship between fatigue and low processing fluency can be understood as an interaction between the body and the brain. When the body is fatigued, the brain’s inability to process information slows down significantly, resulting in a state of low processing fluency (Anderson and Cockle, 2021). At the same time, when the brain excessive reduction of information processing time, it can also lead to physical fatigue, the feeling of fatigue (Mizuno et al., 2011). This interaction results in a strong partial correlation. A balanced diet and sleep to enhance physical and brain health will help relieve fatigue and low processing fluency conditions. The predictability of different nodes varies considerably, from 39% (Working memory loss) to 60% (Emotion regulation disorder), with an average value of 55%, indicating that all adjacent nodes in the network account for 55% of the average node variance. This result suggests that the nine dimensions of ego depletion in Chinese male young adults are more likely to be self-determined. In addition, “Emotion regulation disorder” is highly predictable, indicating that this dimension is strongly influenced by adjacent nodes in the network. This may provide some important insights, as it suggests that interventions for “Emotion regulation disorder” can also be focused on its powerful neighbors (Social withdrawal and Work burnout). Of all the nodes, “Working memory loss” and “Emotion regulation disorder” are the least and the most predictable, respectively. It should be noted that predictability is an upper-bound estimate.

The edge results show that the strongest edges are between “Somatic distress” and “Fatigue,” “Emotion regulation disorder” and “Social withdrawal,” “Work burnout” and “Low self-efficacy,” “Low adherence” and “Low self-efficacy,” and “Fatigue” and “Low processing fluency.” “Fatigue,” “Low self-efficacy” and “Emotion regulation disorder” have the highest strength centrality in the ego depletion network, indicating that these three nodes are the most strongly associated with other nodes in the network. The results suggest that “Fatigue,” “Low self-efficacy” and “Emotion regulation disorder” play a key role in the formation and stability of ego depletion aftereffect networks. “Fatigue,” “Low self-efficacy” and “Emotion regulation disorder” interact to form a stable network structure, which makes individuals more likely to fall into a cycle of ego depletion in the face of difficulties and challenges. It is important to note that these three factors have no direct connections in the ego depletion aftereffect network, so we speculate that the form of interaction between them may not be direct, it is more likely to be spread through other factors, such as “Work burnout” “Low adherence,” “Somatic distress” and “Social withdrawal.” According to Borsboom’s network theory, the behavior of a system can be explained by nodes and their interactions (Borsboom et al., 2011). “Fatigue,” “Low self-efficacy” and “Emotion regulation disorder” are the key nodes in the ego depletion aftereffect network. The interaction between these nodes can cause the ego-depleting aftereffect network to turn into a vicious circle structure. This cyclic structure can constantly strengthen itself, resulting in the individual in the face of new challenges in the ego depletion response. Baumeister’s research shows that ego depletion can lead to poorer mental, emotional and behavioral performance (Baumeister et al., 1998). This effect is consistent with the interaction between network “Fatigue,” “Low self-efficacy” and “Emotion regulation disorder.” These influences can have doubt and negative effects on people’s thinking, decision-making and behavior, leading people to the cycle of ego depletion. In summary, “Fatigue” “Low self-efficacy” and “Emotion regulation disorder” are key nodes in the ego depletion aftereffect network, and their interactions can lead to a vicious cycle. This cycle can lead to an ever-increasing ego depletion response in an individual’s life. Therefore, for individuals with these factors, effective intervention should be adopted to help them get rid of the impact of ego depletion, so as to improve their quality of life and efficiency. The design of intervention program should focus on the interaction of factors and the overall network structure to achieve optimal results. The integration of different interventions targeting “Fatigue,” “Low self-efficacy” and “Emotion regulation disorder” may be effective in improving the effectiveness of the interventions. Based on the study results, it can be concluded that the network connectivity of ego depletion is high, and there is a close relationship between nodes.

There are some limitations of this study. First, the network structure constructed in this paper studies the inter-agent effect of a group, indicating that the network structure may not be replicable for a single individual. Second, network analysis is only one way to describe the relationships between observed variables, and the results are sample-specific. The study has focused on “subclinical” manifestations of the state of low self-control, mainly in male college students at a science and engineering university. It would be worthwhile to examine whether these network structures replicate in samples with higher rates of malevolent behavior, such as criminal offenders. It is also useful to check the consistency of these network structures in a particular environment, such as a high competition or stressful environment. Third, because we used cross-sectional data for analysis, we cannot determine the direction of the edge. For example, we cannot explain whether the central node activates other nodes, is activated by other nodes, or both. The relationships discussed here cannot be considered causal and require longitudinal data analysis. The empirical sampling method (ESM), as an intensive longitudinal data collection method, repeated measurements of symptoms in real-life situations with the help of cues from some devices, such as computers and smartphones, data collection closer to the nature of psychiatric symptoms. In recent years, the ESM has become increasingly popular in mental health because of its ability to capture variables over time (Myin-Germeys et al., 2018). Future studies can use the ESM to explore the temporal causality of these variables and to obtain a personalized network model with customized interventions.

## Data availability statement

The original contributions presented in the study are included in the article/Supplementary material, further inquiries can be directed to the corresponding authors.

## Ethics statement

The studies involving human participants were reviewed and approved by the Ethics Committee of the Air Force Medical University. The patients/participants provided their written informed consent to participate in this study.

## Author contributions

JY, LR, and XL conceived and designed the study. JY, WX, and JZ collected the data. JY and LR analyzed the data. LR, JY, YZ, and XL contributed reagents, materials, and analysis tools. JY, LR, XZ, WX, and XL wrote the manuscript. All authors contributed to the article and approved the submitted version.

## Funding

XL’s involvement in this research was funded by the Year 2022 Major Projects of Military Logistic Research Grant and the Key Project of Air Force Equipment Comprehensive Research (KJ2022A000415).

## Conflict of interest

The authors declare that the research was conducted in the absence of any commercial or financial relationships that could be construed as a potential conflict of interest.

## Publisher’s note

All claims expressed in this article are solely those of the authors and do not necessarily represent those of their affiliated organizations, or those of the publisher, the editors and the reviewers. Any product that may be evaluated in this article, or claim that may be made by its manufacturer, is not guaranteed or endorsed by the publisher.

## Supplementary material

The Supplementary material for this article can be found online at: https://www.frontiersin.org/articles/10.3389/fpsyg.2023.1102624/full#supplementary-material
