# Supplementary material for: The network structure of ego depletion in Chinese male young adults
Source: Front Psychol. 2023 May 17;14:1102624. doi: 10.3389/fpsyg.2023.1102624 (PMC10231657; doi:10.3389/fpsyg.2023.1102624)
Supplement: Supplementary file 1 [file Data_Sheet_1.docx]

**Supplementary Materials**

1. Table S1. Nonparametric Spearman rho correlation matrix of dimensions in the present network
2. Figure S1. Accuracy of edge weight
3. Figure S2. Bootstrapped difference test for edge weight
4. Figure S3. Stability of node strength centrality
5. Figure S4. Bootstrapped difference test for node strength centrality

Table S1. Nonparametric Spearman rho correlation matrix of dimensions in the present network

|  | SD | FA | LPF | WB | WML | ERD | SW | LA | LSE |
| --- | --- | --- | --- | --- | --- | --- | --- | --- | --- |
| Somatic distress (SD) | 1.00 |  |  |  |  |  |  |  |  |
| Fatigue (FA) | 0.66^*^ | 1.00 |  |  |  |  |  |  |  |
| Low processing fluency (LPF) | 0.55^*^ | 0.63^*^ | 1.00 |  |  |  |  |  |  |
| Work burnout (WB) | 0.56^*^ | 0.61^*^ | 0.61^*^ | 1.00 |  |  |  |  |  |
| Working memory loss (WML) | 0.42^*^ | 0.41^*^ | 0.54^*^ | 0.46^*^ | 1.00 |  |  |  |  |
| Emotion regulation disorder (ERD) | 0.50^*^ | 0.52^*^ | 0.55^*^ | 0.59^*^ | 0.55^*^ | 1.00 |  |  |  |
| Social withdrawal (SW) | 0.50^*^ | 0.48^*^ | 0.55^*^ | 0.58^*^ | 0.49^*^ | 0.69^*^ | 1.00 |  |  |
| Low adherence (LA) | 0.49^*^ | 0.58^*^ | 0.58^*^ | 0.56^*^ | 0.47^*^ | 0.54^*^ | 0.62^*^ | 1.00 |  |
| Low self-efficacy (LSE) | 0.47^*^ | 0.54^*^ | 0.58^*^ | 0.64^*^ | 0.54^*^ | 0.54^*^ | 0.63^*^ | 0.64^*^ | 1.00 |

^*^ *p* < 0.01 (two-tailed)

Figure S1. Accuracy of edge weight

*Note*: The red line depicts the sample edge weight and the gray bar depicts the bootstrapped confidence interval.

Figure S2. Bootstrapped difference test for edge weight

*Note*: Gray boxes indicate edge weight that do not differ significantly from one another, while black boxes indicate edge weight that do differ significantly. Blue and red boxes on the diagonal correspond to edge weight with positive and negative correlations, respectively.

Figure S3. Stability of node strength centrality

*Note*: The red bar represents the average correlation between node strength centrality in the full sample and subsample with the red area depicting the 2.5th quantile to the 97.5th quantile.

Figure S4. Bootstrapped difference test for node strength centrality

*Note*: Gray boxes indicate node strength centrality that do not differ significantly from one another, while black boxes indicate node strength centrality that do differ significantly. The number in the white boxes (i.e., diagonal line) represent the value of node strength centrality.


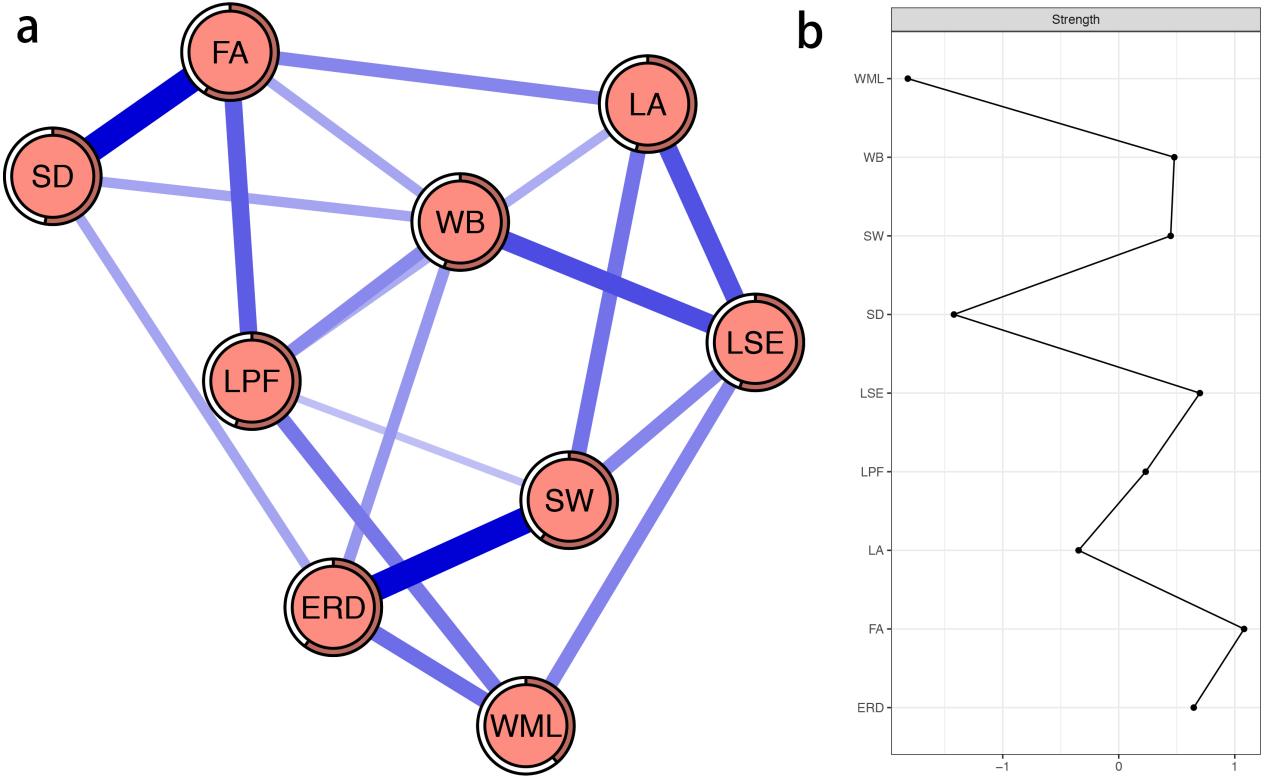


Figure 1a. EDS-A network. Somatic distress (SD) , fatigue (FA) , low processing fluency (LPF) , work burnout (WB) , working memory loss (WML) , emotional regulation disorder (ERD) , social withdrawal (SW) , low adherence (LA) , and low self-efficacy (LSE) . The thickness of the edges represents the degree of correlation. The blue edge represents a positive correlation, and the red edge represents a negative correlation. The rings around the nodes describe its predictability.

Figure 1b. Strength of Z-scores of each node in EDS-A network. Somatic distress (SD) , fatigue (FA) , low processing fluency (LPF) , work burnout (WB) , working memory loss (WML) , emotional regulation disorder (ERD) , social withdrawal (SW) , low adherence (LA) , and low self-efficacy (LSE) .
